# The number of tree species on Earth

**DOI:** 10.1073/pnas.2115329119

**Published:** 2022-01-31

**Authors:** Roberto Cazzolla Gatti, Peter B. Reich, Javier G. P. Gamarra, Tom Crowther, Cang Hui, Albert Morera, Jean-Francois Bastin, Sergio de-Miguel, Gert-Jan Nabuurs, Jens-Christian Svenning, Josep M. Serra-Diaz, Cory Merow, Brian Enquist, Maria Kamenetsky, Junho Lee, Jun Zhu, Jinyun Fang, Douglass F. Jacobs, Bryan Pijanowski, Arindam Banerjee, Robert A. Giaquinto, Giorgio Alberti, Angelica Maria Almeyda Zambrano, Esteban Alvarez-Davila, Alejandro Araujo-Murakami, Valerio Avitabile, Gerardo A. Aymard, Radomir Balazy, Chris Baraloto, Jorcely G. Barroso, Meredith L. Bastian, Philippe Birnbaum, Robert Bitariho, Jan Bogaert, Frans Bongers, Olivier Bouriaud, Pedro H. S. Brancalion, Francis Q. Brearley, Eben North Broadbent, Filippo Bussotti, Wendeson Castro da Silva, Ricardo Gomes César, Goran Češljar, Víctor Chama Moscoso, Han Y. H. Chen, Emil Cienciala, Connie J. Clark, David A. Coomes, Selvadurai Dayanandan, Mathieu Decuyper, Laura E. Dee, Jhon Del Aguila Pasquel, Géraldine Derroire, Marie Noel Kamdem Djuikouo, Tran Van Do, Jiri Dolezal, Ilija Đ. Đorđević, Julien Engel, Tom M. Fayle, Ted R. Feldpausch, Jonas K. Fridman, David J. Harris, Andreas Hemp, Geerten Hengeveld, Bruno Herault, Martin Herold, Thomas Ibanez, Andrzej M. Jagodzinski, Bogdan Jaroszewicz, Kathryn J. Jeffery, Vivian Kvist Johannsen, Tommaso Jucker, Ahto Kangur, Victor N. Karminov, Kuswata Kartawinata, Deborah K. Kennard, Sebastian Kepfer-Rojas, Gunnar Keppel, Mohammed Latif Khan, Pramod Kumar Khare, Timothy J. Kileen, Hyun Seok Kim, Henn Korjus, Amit Kumar, Ashwani Kumar, Diana Laarmann, Nicolas Labrière, Mait Lang, Simon L. Lewis, Natalia Lukina, Brian S. Maitner, Yadvinder Malhi, Andrew R. Marshall, Olga V. Martynenko, Abel L. Monteagudo Mendoza, Petr V. Ontikov, Edgar Ortiz-Malavasi, Nadir C. Pallqui Camacho, Alain Paquette, Minjee Park, Narayanaswamy Parthasarathy, Pablo Luis Peri, Pascal Petronelli, Sebastian Pfautsch, Oliver L. Phillips, Nicolas Picard, Daniel Piotto, Lourens Poorter, John R. Poulsen, Hans Pretzsch, Hirma Ramírez-Angulo, Zorayda Restrepo Correa, Mirco Rodeghiero, Rocío Del Pilar Rojas Gonzáles, Samir G. Rolim, Francesco Rovero, Ervan Rutishauser, Purabi Saikia, Christian Salas-Eljatib, Dmitry Schepaschenko, Michael Scherer-Lorenzen, Vladimír Šebeň, Marcos Silveira, Ferry Slik, Bonaventure Sonké, Alexandre F. Souza, Krzysztof Jan Stereńczak, Miroslav Svoboda, Hermann Taedoumg, Nadja Tchebakova, John Terborgh, Elena Tikhonova, Armando Torres-Lezama, Fons van der Plas, Rodolfo Vásquez, Helder Viana, Alexander C. Vibrans, Emilio Vilanova, Vincent A. Vos, Hua-Feng Wang, Bertil Westerlund, Lee J. T. White, Susan K. Wiser, Tomasz Zawiła-Niedźwiecki, Lise Zemagho, Zhi-Xin Zhu, Irié C. Zo-Bi, Jingjing Liang

**Affiliations:** ^a^Department of Forestry and Natural Resources, Purdue University, West Lafayette, IN 47907;; ^b^Department of Biological, Geological, and Environmental Sciences, Alma Mater Studiorum University of Bologna, Bologna 40126, Italy;; ^c^Biological Institute, Tomsk State University, Tomsk 634050, Russia;; ^d^Department of Forest Resources, University of Minnesota, St. Paul, MN 55108;; ^e^Institute for Global Change Biology and School for Environment and Sustainability, University of Michigan, Ann Arbor, MI 48109;; ^f^Hawkesbury Institute for the Environment, Western Sydney University, Penrith 2753, Australia;; ^g^Forestry Department, FAO, Rome 00153, Italy;; ^h^Institute of Integrative Biology, ETH Zurich 8092 Zurich, Switzerland;; ^i^Centre for Invasion Biology, Department of Mathematical Sciences, Stellenbosch University, Stellenbosch 7602, South Africa;; ^j^Mathematical Biology Unit, African Institute for Mathematical Sciences, Muizenberg 7945, South Africa;; ^k^Department of Crop and Forest Sciences, University of Lleida 25198 Lleida, Spain;; ^l^Joint Research Unit CTFC–AGROTECNIO–CERCA 25280 Solsona, Spain;; ^m^TERRA Teaching and Research Centre, Gembloux Agro-Bio Tech, University of Liege, Gembloux 5030, Belgium;; ^n^Forest Ecology and Forest Management Group, Wageningen University and Research, Wageningen 6700 AA, The Netherlands;; ^o^Center for Biodiversity Dynamics in a Changing World (BIOCHANGE), Department of Biology, Aarhus University DK-8000 Aarhus C, Denmark;; ^p^Section for Ecoinformatics and Biodiversity, Department of Biology, Aarhus University DK-8000 Aarhus C, Denmark;; ^q^AgroParisTech, INRAE, Silva, Université de Lorraine 5400 Nancy, France;; ^r^Department of Ecology and Evolutionary Biology, University of Connecticut, Mansfield, CT 06268;; ^s^Department of Ecology and Evolutionary Biology, University of Arizona, Tucson, AZ 85721;; ^t^Department of Population Health Sciences, University of Wisconsin–Madison, Madison, WI 53704;; ^u^Statistics Program, King Abdullah University of Science and Technology, Thuwal 23955, Saudi Arabia;; ^v^Department of Statistics, University of Wisconsin–Madison, Madison, WI 53706;; ^w^Institute of Botany, CAS, Peking University, Beijing 100871, China;; ^x^Department of Computer Science, University of Illinois at Urbana–Champaign, IL 61801;; ^y^Department of Computer Science and Engineering, University of Minnesota, Minneapolis, MN 55108;; ^z^Department of Agricultural, Food, Environmental and Animal Sciences, University of Udine 33100, Italy;; ^aa^Faculty of Science and Technology, Free University of Bolzano-Bozen, Bolzano 39100, Italy;; ^bb^Spatial Ecology and Conservation Lab, Department of Tourism, Hospitality, and Events Management, University of Florida, Gainesville, FL 32611;; ^cc^Escuela de Ciencias Ambientales, Universidad Nacional (Colombia) Abierta y a Distancia, Bogotá 2102, Colombia;; ^dd^Museo de Historia Natural Noel Kempff Mercado, Universidad Autónoma Gabriel Rene Moreno, Casilla 2489, Santa Cruz, Bolivia;; ^ee^European Commission, Joint Research Centre 21027 Ispra, Italy;; ^ff^Compensation International Progress S. A.–Ciprogress Greenlife, Bogotá, Colombia;; ^gg^UNELLEZ-Guanare, Programa de Ciencias del Agro y el Mar, Herbario Universitario (PORT), Mesa de Cavacas, estado Portuguesa 3323, Venezuela;; ^hh^Department of Geomatics, Forest Research Institute, Sękocin Stary, Raszyn 05-090, Poland;; ^ii^International Center for Tropical Botany, Department of Biological Sciences, Florida International University, Miami, FL 33133;; ^jj^Forest Science Laboratory, Multidisciplinary Center, Universidade Federal do Acre, Cruzeiro do Sul 69920-900, Brazil;; ^kk^Proceedings of the National Academy of Sciences, U.S.A., Washington, DC 20001;; ^ll^Department of Evolutionary Anthropology, Duke University, Durham, NC 27708-06080;; ^mm^AMAP, Université de Montpellier, CIRAD, CNRS, INRAE, IRD, Montpellier 34090, France;; ^nn^Institut Agronomique néo-Calédonien, Equipe Sol & Végétation 98800 Noumea, New Caledonia;; ^oo^Institute of Tropical Forest Conservation, Mbarara University of Science and Technology, Kabale, Uganda;; ^pp^Integrated Center for Research, Development and Innovation in Advanced Materials, Nanotechnologies, and Distributed Systems for Fabrication and Control, University Stefan cel Mare of Suceava 720229 Suceava, Romania;; ^qq^Department of Forest Sciences, “Luiz de Queiroz” College of Agriculture, University of São Paulo, Piracicaba 13418-900, Brazil;; ^rr^Department of Natural Sciences, Manchester Metropolitan University, Manchester M1 5GD, United Kingdom;; ^ss^Spatial Ecology and Conservation Lab, School of Forest Resources and Conservation, University of Florida, Gainesville, FL 32611;; ^tt^Department of Agriculture, Alimentation, Environment and Forestry, Università degli Studi di Firenze, Firenze 50144, Italy;; ^uu^Amazonia Green Landscape Protection and Governance Programme, SOS Amazônia, Rio Branco 69905-082, Brazil;; ^vv^Laboratory of Botany and Plant Ecology, Center of Biological and Nature Sciences, Federal University of Acre, Rio Branco 69920-900, Brazil;; ^ww^Department of Spatial Regulation, GIS and Forest Policy, Institute of Forestry 11030 Belgrade, Serbia;; ^xx^Universidad Nacional de San Antonio Abad del Cusco, Cusco 08000, Peru;; ^yy^Faculty of Natural Resources Management, Lakehead University, Thunder Bay, ON, Canada P7B 5E1;; ^zz^Institute of Forest Ecosystem Research 254 01 Jilove u Prahy, Czech Republic;; ^aaa^Global Change Research Institute of the Czech Academy of Sciences 603 00 Brno, Czech Republic;; ^bbb^Nicholas School of the Environment, Duke University, Durham, NC 27708;; ^ccc^Department of Plant Sciences in the Conservation Research Institute, University of Cambridge, Cambridge CB2 3EA, United Kingdom;; ^ddd^Quebec Center for Biodiversity Sciences, Centre for Sustainability Research, CSFG and Biology Department, Concordia University, Montreal, ON QC H3G 1M8, Canada;; ^eee^Laboratory for Geoinformation Science and Remote Sensing, Department of Environmental Sciences, Wageningen University and Research, Wageningen 6700 AA, The Netherlands;; ^fff^World Agroforestry (ICRAF), Nairobi 00100, Kenya;; ^ggg^Ecology and Evolutionary Biology, University of California, Santa Barbara, CA 93106;; ^hhh^Instituto de Investigaciones de la Amazonia Peruana, Iquitos, Peru;; ^iii^Cirad, UMREcoFoG (Agroparistech, CNRS, INRAE, Université des Antilles, Université de Guyane) 97 310 Kourou, French Guiana;; ^jjj^Department of Plant Science, Faculty of Science, University of Buea, Buea, Cameroon;; ^kkk^Silviculture Rearch Institute, Vietnamese Academy of Forest Sciences, Hanoi, Vietnam;; ^lll^Institute of Botany of the Czech Academy of Science 25243 Průhonice, Czech Republic;; ^mmm^Faculty of Science, University of South Bohemia 37005 České Budějovice, Czech Republic;; ^nnn^AMAP, IRD, CIRAD, CNRS, INRAE, Université de Montpellier, Montpellier Cedex 5 F-34398, France;; ^ooo^School of Biological and Behavioural Sciences, Queen Mary University of London, London E1 4NS, United Kingdom;; ^ppp^College of Life and Environmental Sciences, University of Exeter, Exeter EX4 4PY, United Kingdom;; ^qqq^Department of Forest Resource Management, Swedish University of Agricultural Sciences, Uppsala 750 07, Sweden;; ^rrr^Royal Botanic Garden Edinburgh, Edinburgh EH3 5LR, United Kingdom;; ^sss^Department of Plant Systematics, University of Bayreuth 95440 Bayreuth, Germany;; ^ttt^Biometris & Forest and Nature Policy Group, Wageningen University and Research, Wageningen 6700 AA, The Netherlands;; ^uuu^CIRAD, UPR Forêts et Sociétés, Yamoussoukro, Côte d’Ivoire;; ^vvv^Forêts et Sociétés, Univ Montpellier, CIRAD, Montpellier 34000, France;; ^www^Institut National Polytechnique Félix Houphouët-Boigny, Yamoussoukro, Côte d’Ivoire;; ^xxx^Laboratory for Geoinformation Science and Remote Sensing, Department of Environmental Sciences, Wageningen University and Research, Wageningen 6700 AA, The Netherlands;; ^yyy^Section 1.4 Remote Sensing and Geoinformatics, Helmholtz GFZ German Research Centre for Geosciences, Telegrafenberg, Potsdam 14473, Germany;; ^zzz^Institut Agronomique néo-Calédonien, Equipe Sol & Végétation 98800, New Caledonia;; ^aaaa^Department of Biology, University of Hawai'i at Hilo, Hilo, HI 96720;; ^bbbb^Institute of Dendrology, Polish Academy of Sciences, Kornik 62-035, Poland;; ^cccc^Białowieża Geobotanical Station, Faculty of Biology, University of Warsaw, Warsaw 17-230, Poland;; ^dddd^Faculty of Natural Sciences, University of Stirling, Stirling FK9 4LA, United Kingdom;; ^eeee^Department of Geosciences and Natural Resource Management, University of Copenhagen 1165, Denmark;; ^ffff^School of Biological Sciences, University of Bristol, Bristol BS8 1TQ, United Kingdom;; ^gggg^Institute of Forestry and Rural Engineering, Estonian University of Life Sciences, Tartu 51006, Estonia;; ^hhhh^Center for Forest Ecology and Productivity of the Russian Academy of Sciences, Moscow 119991, Russia;; ^iiii^Integrative Research Center, The Field Museum, Chicago, IL 60605;; ^jjjj^Herbarium Bogoriense, Research Center for Biology, Indonesian Institute of Sciences/Lembaga Ilmu Pengetahuan Indonesia, Cibinong Science Center, Cibinong 16912 Indonesia;; ^kkkk^Department of Physical and Environmental Sciences, Colorado Mesa University, Grand Junction, CO 81501-3122;; ^llll^Department of Geosciences and Natural Resource Management, University of Copenhagen, Copenhagen 1017, Denmark;; ^mmmm^UniSA STEM and Future Industries Institute, University of South Australia 5001 Adelaide, Australia;; ^nnnn^Department of Botany, Dr. Harisingh Gour Vishwavidyalaya (A Central University), Sagar 470003, India;; ^oooo^Department of Botany, Dr. Harisingh Gour Central University, Madhya Pradesh Sagar-470003, India;; ^pppp^Museo de Historia natural Noel Kempff Mercado, Santa Cruz, Bolivia;; ^qqqq^Department of Agriculture, Forestry and Bioresources, Seoul National University, Seoul 08826, South Korea;; ^rrrr^Interdisciplinary Program in Agricultural and Forest Meteorology, Seoul National University, Seoul 08826, South Korea;; ^ssss^National Center for AgroMeteorology, Seoul 08826, South Korea;; ^tttt^Institute of Future Environmental and Forest Resources, Research Institute for Agriculture and Life Sciences, Seoul National University, Seoul 08826, South Korea;; ^uuuu^Department of Geoinformatics, Central University of Jharkhand, Ranchi 835205, Jharkhand, India;; ^vvvv^Department of Botany, Dr. Harisingh Gour Vishwavidyalaya (A Central University), Sagar 470003, India;; ^wwww^Laboratoire Évolution et Diversité Biologique, UMR 5174 (CNRS/IRD/UPS) 31062 Toulouse Cedex 9, France;; ^xxxx^Tartu Observatory, University of Tartu, Tartu 61602, Estonia;; ^yyyy^School of Geography, University of Leeds, Leeds LS2 9JT, United Kingdom;; ^zzzz^Department of Geography, University College London, London WC1E 6BT, United Kingdom;; ^aaaaa^Department of Ecology and Evolutionary Biology, University of Arizona, Tucson, AZ 85721;; ^bbbbb^Environmental Change Institute, School of Geography and the Environment, University of Oxford, Oxford OX1 3QY, United Kingdom;; ^ccccc^Forest Research Institute, University of the Sunshine Coast, Sippy Downs QLD 4556, Australia;; ^ddddd^Department of Environment and Geography, University of York YO10 5NG, United Kingdom;; ^eeeee^All-Russian Institute of Continuous Education in Forestry 141200 Pushkino, Moscow region, Russia;; ^fffff^Universidad Nacional de San Antonio Abad del Cusco, Cusco, Peru;; ^ggggg^FSBI “ROSLESINFORG,” 141208 Ivanteevka, Moscow region, Russia;; ^hhhhh^Escuela de Ingeniería Forestal, Insttuto Tecnologico de Costa Rica, Cartago 30101, Costa Rica;; ^iiiii^Universidad Nacional de San Antonio Abad del Cusco, Cusco, Peru;; ^jjjjj^Département des sciences biologiques, Centre for Forest Research, Université du Québec à Montreal, Montreal, QC, Canada H3C 3P8;; ^kkkkk^Department of Ecology and Environmental Sciences, Pondicherry University, Puducherry 605 014, India;; ^lllll^Instituto Nacional de Tecnologia Agropecuaria–Universidad Nacional de la Patagonia Austral–CONICET, Río Gallegos CP 9400, Argentina;; ^mmmmm^Cirad, UMREcoFoG (Agroparistech, CNRS, INRAE, Université des Antilles, Université de Guyane) 97 310 Kourou, French Guiana;; ^nnnnn^Urban Ecosystem Research, School of Social Science, Western Sydney University, Penrith, NSW 2751, Australia;; ^ooooo^Groupement d’Intérêt Public (GIP) Ecofor, Paris 75116, France;; ^ppppp^Laboratory of Tropical Dendrology and Forestry, Training Center in Agroforestry Sciences, Federal University of Southern Bahia, Ilheus 45613-204, Brazil;; ^qqqqq^School of Life Sciences, Chair of Forest Growth and Yield Science, Technical University of Munich 85354 Freising, Germany;; ^rrrrr^Instituto de Investigaciones paa el Desarrollo Forestal, Universidad de Los Andes, Mérida 5101, Venezuela;; ^sssss^Escuela Ambiental, Facultad de Ingeniería, Universidad de Antioquia, Medellin, Colombia;; ^ttttt^Agriculture Food Environment Centre (C3A), University of Trento, San Michele all’Adige 38122, Italy;; ^uuuuu^Research and Innovation Centre, Fondazione Edmund Mach, San Michele all’Adige 38010, Italy;; ^vvvvv^Herbario Selva Central (HOXA), Jardín Botánico de Missouri, Oxapampa, Pasco Mz.E-6, Peru;; ^wwwww^Laboratory of Tropical Dendrology and Forestry, Training Center in Agroforestry Sciences, Federal University of Southern Bahia, Ilheus 45613-204, Brazil;; ^xxxxx^Department of Biology, University of Florence 50019 Sesto Fiorentino, Italy;; ^yyyyy^MUSE-Museo delle Scienze 38122 Trento, Italy;; ^zzzzz^InfoFlora, Conservatoire et Jardin Botanique de Genève 1292 Chambesy, Switzerland;; ^aaaaaa^Department of Environmental Sciences, Central University of Jharkhand, Ranchi 835205, India;; ^bbbbbb^Centro de Modelacion y Monitoreo de Ecosistemas, Universidad Mayor, Universidad de La Frontera, Santiago, Chile;; ^cccccc^Vicerrectoría de Investigación y Postgrado, Universidad de La Frontera, Temuco 4811230, Chile;; ^dddddd^Departamento de Silvicultura y Cons. de la Naturaleza, Universidad de Chile, Santiago 8820808, Chile;; ^eeeeee^Sukachev Institute of Forest of the SB Russian Academy of Sciences 660036 Krasnoyarsk, Russia;; ^ffffff^International Institute for Applied Systems Analysis, Laxenburg A-2361, Austria;; ^gggggg^Faculty of Biology, Geobotany, University of Freiburg 79104 Freiburg, Germany;; ^hhhhhh^National Forest Centre 96001 Zvolen, Slovakia;; ^iiiiii^Laboratory of Botany and Plant Ecology, Center of Biological and Nature Sciences, Federal University of Acre, Rio Branco 69920-900, Brazil;; ^jjjjjj^Environmental and Life Sciences, Faculty of Science, Universiti Brunei Darussalam BE1410 Gadong, Brunei;; ^kkkkkk^Plant Systematic and Ecology Laboratory, Department of Biology, Higher Teachers' Training College, University of Yaounde I, Yaounde, Cameroon;; ^llllll^CB, Departamento de Ecologia, Universidade Federal do Rio Grande do Norte, Natal 59072-970, Brazil;; ^mmmmmm^Department of Geomatics, Forest Research Institute, Sękocin Stary, Raszyn 05-090, Poland;; ^nnnnnn^Faculty of Forestry and Wood Sciences, Czech University of Life Sciences Prague 16521 Prague, Czech Republic;; ^oooooo^Department of Plant Biology, Faculty of Sciences, University of Yaounde I, Yaounde, Cameroon;; ^pppppp^Bioversity International, IITA Regional Office in Cameroon, Yaounde, Cameroon;; ^qqqqqq^Department of Biology and Florida Museum of Natural History, University of Florida, Gainesville, FL 32611;; ^rrrrrr^School of Science and Engineering, James Cook University, Cairns QLD 4878, Australia;; ^ssssss^Instituto de Investigaciones paa el Desarrollo Forestal, Universidad de Los Andes, Mérida 5101, Venezuela;; ^tttttt^Systematic Botany and Functional Biodiversity, Institute of Biology, Leipzig University 04103 Leipzig, Germany;; ^uuuuuu^Centre for the Research and Technology of Agro-Environmental and Biological Sciences, CITAB, University of Trás-os-Montes and Alto Douro, UTAD, Quinta de Prados 5000-801 Vila Real, Viseu, Portugal;; ^vvvvvv^Department of Ecology and Sustainable Agriculture, Agricultural High School, Polytechnic Institute of Viseu 3500-606 Viseu, Portugal;; ^wwwwww^Department of Forest Engineering, Universidade Regional de Blumenau, Blumenau-Santa Catarina 89030, Brazil;; ^xxxxxx^Department of Environmental Science, Policy, and Management, University of California, Berkeley, CA 94720;; ^yyyyyy^Universidad Autónoma del Beni, Riberalta, Beni 2W3Q+VHJ, Bolivia;; ^zzzzzz^Key Laboratory of Tropical Biological Resources of Ministry of Education, School of Life and Pharmaceutical Sciences, Hainan University, Haikou 570228, China;; ^aaaaaaa^Department of Forest Resource Management, Swedish University of Agricultural Sciences, Umeå 750 07, Sweden;; ^bbbbbbb^Ministere des Eaux, des Forets, de la Mer et de l’Environnement chargé du Plan Climat et Objectifs de Development Durable, Estuaire Libreville, Gabon;; ^ccccccc^Institut de Recherche en Ecologie Tropicale, Libreville, Gabon;; ^ddddddd^Faculty of Natural Sciences, University of Stirling FK9 4LA, United Kingdom;; ^eeeeeee^Manaaki Whenua–Landcare Research, Lincoln 7640, New Zealand;; ^fffffff^State Forests–Coordination Centre for Environmental Projects, Warsaw 02-362, Poland;; ^ggggggg^Key Laboratory of Tropical Biological Resources of Ministry of Education, School of Life and Pharmaceutical Sciences, Hainan University, Haikou 570228, China;; ^hhhhhhh^Département FOREN, Institut National Polytechnique Félix Houphouët-Boigny, Yamoussoukro BP 1093, Côte d’Ivoire

**Keywords:** biodiversity, forests, hyperdominance, rarity, richness

## Abstract

One of the most fundamental questions in ecology is how many species inhabit the Earth. However, due to massive logistical and financial challenges and taxonomic difficulties connected to the species concept definition, the global numbers of species, including those of important and well-studied life forms such as trees, still remain largely unknown. Here, based on global ground-sourced data, we estimate the total tree species richness at global, continental, and biome levels. Our results indicate that there are ∼73,000 tree species globally, among which ∼9,000 tree species are yet to be discovered. Roughly 40% of undiscovered tree species are in South America. Moreover, almost one-third of all tree species to be discovered may be rare, with very low populations and limited spatial distribution (likely in remote tropical lowlands and mountains). These findings highlight the vulnerability of global forest biodiversity to anthropogenic changes in land use and climate, which disproportionately threaten rare species and thus, global tree richness.

## Significance

Tree diversity is fundamental for forest ecosystem stability and services. However, because of limited available data, estimates of tree diversity at large geographic domains still rely heavily on published lists of species descriptions that are geographically uneven in coverage. These limitations have precluded efforts to generate a global perspective. Here, based on a ground-sourced global database, we estimate the number of tree species at biome, continental, and global scales. We estimated a global tree richness (≈73,300) that is ≈14% higher than numbers known today, with most undiscovered species being rare, continentally endemic, and tropical or subtropical. These results highlight the vulnerability of global tree species diversity to anthropogenic changes.

In 1994, Robert May (1) provided the optimistic observation that, by 2044, we would roughly know the current number of species on Earth. Half of that time period has already lapsed, and we are still far from that goal. Even for trees, which are among the largest and most widespread organisms on the planet (2–6), provide a wealth of ecosystem services for humans (7–9), and support much of terrestrial biodiversity (10), we still lack a fundamental understanding of how many species exist on our planet (3, 4, 11–13).

A growing body of evidence highlights details and mechanisms regarding the biogeographic patterns in tree species diversity, such as the number of species increasing consistently toward equatorial regions (14–16). With a manageable number of taxa, tree species in the higher latitudes are relatively well characterized. However, if hyperdominance of a small fraction of species in the tropics (17) is a general phenomenon, it would mean that these regions generally harbor a very large number of rare species, many of which are endemic. The contribution of rare species to ecosystem services may be relevant and is a topic of active research (18, 19), but it is challenging as most remain poorly documented (20–26). Therefore, estimating the number of tree species is essential to inform, optimize, and prioritize forest conservation efforts across the globe. Knowing diversity’s extents will be useful in several ways. First, it can help us to infer the evolutionary mechanisms that have generated diversity, so that we can predict how those same mechanisms may play out in the future. Second, it may assist in assessment of which systems may be most resilient to global change. Third, if undetected species are mostly rare and rare species are more vulnerable to extinction risk, having a better grasp of those numbers is essential to managing for biodiversity preservation. Finally, with an understanding of total species pools, it is possible to quantify the impacts of regional conservation efforts while also improving the ability to predict extinctions, manage diversity hotspots, or collect germplasm (22, 23).

Because of the limited extent of data available, estimates of tree species diversity in large geographic domains still rely heavily on expert opinions and compiled published lists of species descriptions that are geographically uneven in coverage (24, 25). Although local specialists have been increasingly joining efforts to consolidate species lists in many domains, these limitations have precluded efforts to scale this information to generate a global perspective. Here, based on a ground-sourced global database numbering ∼64,100 species [a value similar to a prior enumeration of the total of known tree species of ∼60,000 (17)], we developed estimates of the number of tree species at biome, continental, and global scales. Specifically, by comparing species accumulation curves (SACs) of tree species across different spatial scales, we estimated the number of species that have not been recorded in the global data compilation used herein.

## Results and Discussion

### Global-, Continental-, and Biome-Level Patterns.

**Fig. 1.**
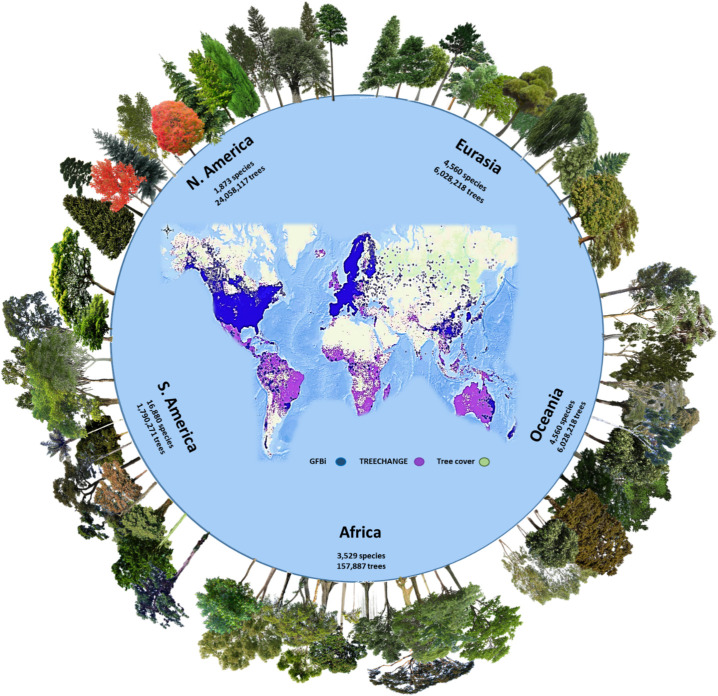
The number of tree species and individuals per continent in the GFBI database. This dataset (blue points in the central map) was used for the parametric estimation and merged with the TREECHANGE occurrence-based data (purple points in the central map) to provide the estimates in this study. Green areas represent the global tree cover. GFBI consists of abundance-based records of ∼38 million trees for 28,192 species. Depicted here are some of the most frequent species recorded in each continent. Some GFBI and TREECHANGE points may overlap in the map.

We compiled a comprehensive global occurrence dataset with 9,353 (100- × 100-km) grid cell samples (called “samples” or “sampling units” hereafter of ∼1°) (*Materials and Methods*) by combining an abundance-based tree species dataset (the Global Forest Biodiversity Initiative [GFBI]) (Fig. 1), based on forest plots worldwide and comprising ∼38 million trees for 28,192 species, with a large high-quality occurrence-based dataset (TREECHANGE) that includes forest plots and botanical vouchers (26) (*Materials and Methods*). It is important to note that despite the large number of grid cells, extensive data, and high mean global sample coverage (96.4%) (Table 1), sampling within grid cells in many regions of the world remains very sparse.

**Table 1.** Observed, asymptotic, and adjusted tree species richness and sample coverage at continental and global scales (note that the global value is lower than the sum of the continental ones due to overlapping species among continents [Fig. 4] and due to independent estimators being run for each continent and globally)

Continent	No. of sampling units (∼1° grid cells)	Species (observed)	Sample coverage, %	Chao2 (asymptotic)	95% CI lower Chao2 (asymptotic)	95% CI upper Chao2 (asymptotic)	Chao2_adj_	95% CI lower Chao2_adj_	95% CI upper Chao2_adj_	To be discovered (∼Chao2_adj_ − *S_obs_*)	Hotspot biomes
Global	9,353	64,088	96.4	89,147	89,141	89,152	**73,274**	73,271	73,276	**9,186**	
Africa	1,575	10,441	96.0	14,031	14,028	14,033	**11,875**	11,874	11,877	**1,434**	Tropical /subtropical moist and dry forests, mainly in the Congo River basin
Eurasia	2,896	14,071	94.3	18,311	18,305	18,316	**16,264**	16,262	16,265	**2,193**	Tropical/subtropical moist and dry forests, mainly in Southeast Asia
North America	2,418	8,646	98.6	10,295	10,290	10,299	**11,131**	11,129	11,134	**2,485**	Tropical subtropical moist and dry forests, mainly in Central America
South America	1,461	27,186	95.0	46,738	46,729	46,747	**31,112**	31,110	31,115	**3,926**	Tropical/subtropical forests, grasslands, savannas, and shrublands, mainly in the Amazon River basin and Andean high mountains
Oceania	1,003	6,680	97.4	9,273	9,267	9,277	**8,235**	8,232	8,237	**1,555**	Tropical/subtropical moist forests, mainly in northeast Australia and the Pacific Islands

We also list some biomes that are hotspots of undiscovered species in each continent (*SI Appendix*, Table S2). Bold indicates the number of species to be discovered globally and continentally.

**Fig. 2.**
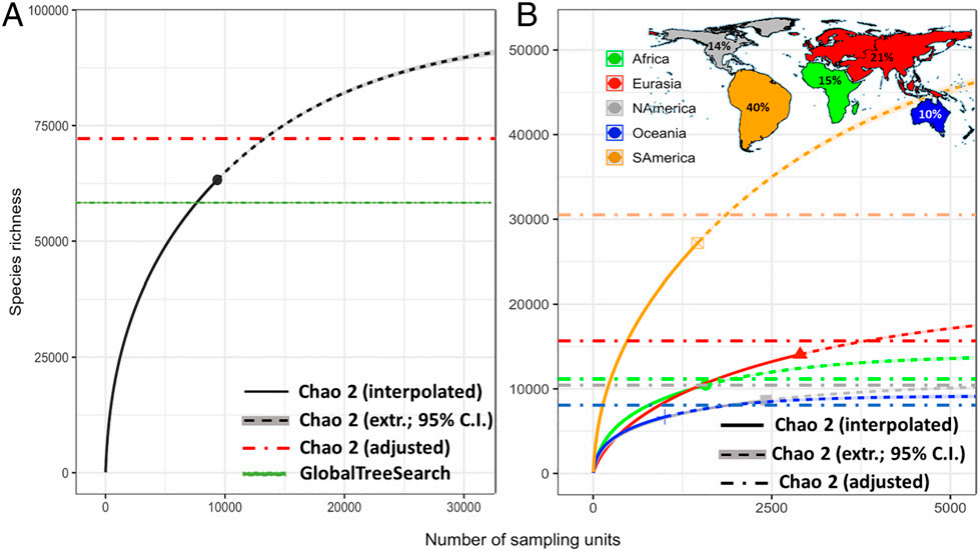
Occurrence-based accumulation curves at global (*A*) and continental (*B*) scales. In *A*, nonparametric (interpolated) and asymptotic (extrapolated) species numbers from Chao2 (upper–lower 95% CI as shaded areas around the means; note that the CI shaded area is narrow because of the high number of sapling units), the Chao2_adj_ estimate for the true number of singletons (red line) vs. the number of samples (1° grid cell ∼100 × 100 km), and the number of species listed in GlobalTreeSearch (green line) are shown. In *B*, nonparametric (interpolated) and asymptotic (extr., extrapolated) estimates (upper–lower 95% CI as shaded areas around the means) and Chao2_adj_ values for the true number of singletons (dashed lines) are displayed vs. the number of samples (1° grid cell ∼100 × 100 km) within continents; the percentage of the global estimated richness in each continent is shown in the cartogram in *B*, *Inset* (total richness per continent is reported in Table 1).

**Fig. 3.**
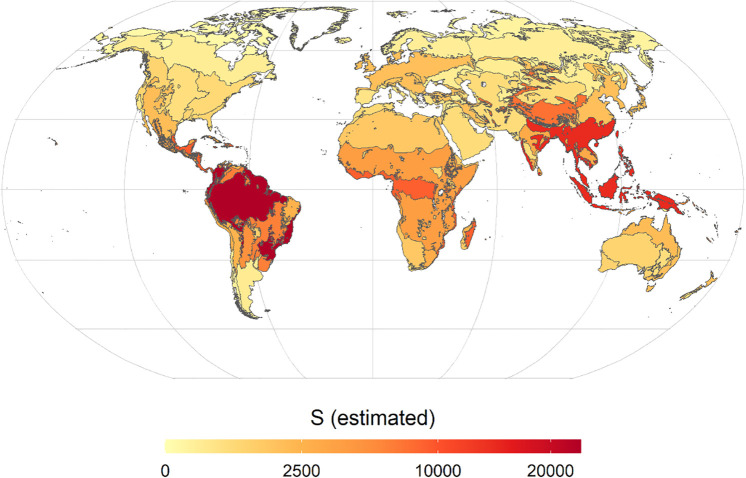
Biome-level tree species richness estimates. The map shows the number of tree species estimated (*S* estimated from Chao2_adj_) in terrestrial biomes of each continent as a color gradient from low richness (yellow) to high richness (red). More information is provided in *SI Appendix*, Table S2.

From this dataset, with a nonparametric estimator [Chao2 (27)], we calculated occurrence-based values of potential global and continental tree species richness (*Materials and Methods*, Fig. 2*A*, and Table 1). This estimator is sensitive to accurate quantification of the numbers of uniques and duplicates (below and *Materials and Methods*), and it is known that there are problems with false uniques in forest species richness datasets (24). These Chao2 values may thus represent an overestimate to the degree that tree species recorded in only one sampling unit have been mistakenly identified as unique. Therefore, we estimated the true number (Chao2_adj_) of unique species (28) (*Materials and Methods*) by accounting for the relationships of uniques, duplicates, triplets, and quadruplets to constrain the estimated numbers of unique species and from this adjusted number, computed a more conservative estimate of global tree richness, which is ∼73,300 species (Chao2_adj_). Based on the good performance of this estimator and its adjusted version reported in previous studies (9, 29–33), we considered the adjusted value (Chao2_adj_) our most reasonable approximation to global tree species richness. We then derived SACs at global (Fig. 2*A*) and continental (Fig. 2*B*) scales. Moreover, for each continent, from the observed number of tree species, we also estimated the asymptotic richness at the within-continent biome-level extent (Fig. 3 and *SI Appendix*, Table S2).

At the global scale, we infer that there likely are ∼9,200 tree species yet to be discovered (Table 1), given the ∼64,000 species already encountered (3, 4, 34–37). Our estimates at continental scales (Fig. 2*B* and Table 1) show that roughly 43% of all Earth’s tree species occur in South America, followed by Eurasia (22%), Africa (16%), North America (15%), and Oceania (11%). However, a lack of saturation (driven by the existence of high numbers of species uncommon in the landscape, incomplete sampling, or both), particularly in the South American accumulation curve (Fig. 2*B*), suggests that our estimates may still be incomplete accounts of continental and global tree species richness. More undiscovered species likely occur in South America than any other continent. Our findings are in general agreement with recent studies of Amazonian plant diversity, which suggested that there are many undiscovered species; moreover, different approaches to the problem arrived at different estimates of total numbers of known and unknown species, suggesting that, as a scientific community, we still have much more work to do to arrive at accurate estimates regionally, continentally, or globally (12, 24, 34–37). Additionally, a considerable number of species have likely not yet been encountered in each of the other four continents as well (Table 1), most likely in the species-rich and more poorly studied tropical regions within each (see below).

Our biome-level estimates of tree richness (Fig. 3 and *SI Appendix*, Table S2) provide a more detailed description of the distribution of species richness within continents and shed more light on South America’s extremely high total tree diversity. As expected, the highest estimates of tree species in all continents are for the tropical and subtropical moist forest biome; for example, roughly half to two-thirds of all already known species occur in these forests on all five continents (*SI Appendix*, Table S2). Moreover, the hotspots of undiscovered species (Table 1 and *SI Appendix*, Table S2) may largely occur in these same species-rich and undersampled (37) regions. However, high numbers of known and unknown species also occur in other biomes, including tropical and subtropical dry forests, temperate forests, mangrove forests, and areas classified as nonforested biomes (e.g., lowland and montane grasslands, savanna, shrublands, deserts) but that include considerable areas of tree-rich, and often speciose, vegetation. The high total tree diversity in South America is dominated by the lowland wet tropics and subtropics, yet roughly one-third of all tree species on that continent are found only outside of that biome.

### Rarity in Forests Worldwide.

We also calculated indices of tree species rarity (percentages of singletons and doubletons) at continental and global scales (*SI Appendix*, Table S3) to help illuminate possible within-sample and among-sample abundance patterns. In fact, since the sample coverage deficit (1 – coverage = slope of the SAC at its right-hand end) is a statistically rigorous way of assessing the incompleteness of sampling (38), the proportion of singletons/uniques is, thus, strongly driven not only by long tails on the underlying species abundance/occurrence distribution but also, by sampling intensity/completeness.

Our most reliable abundance-based asymptotic richness estimates depend on the total number of observed species and the number of species with only one (singletons) or two (doubletons) individuals in each sample (which may represent measures of abundance-based rarity). Similarly, occurrence-based estimates depend on the number of species present in only one (unique) or two (duplicate) samples of each continent (which may represent measures of occurrence-based rarity). Rarity data within samples (*α*; i.e., from the abundance-based dataset) provide an indication of the relative proportion of species that are rare at the landscape to small regional scale represented by individual grid cells (100 × 100 km). The global rarity value is 33%, with Africa (38%) and South America (37%) having the highest percentage of species rare within samples and North America (17%) and Eurasia (24%) having the lowest (*SI Appendix*, Table S3). It is important to note that our data do not mean that one-third of all species occur only once or twice in nature; instead, their rarity in our dataset suggests their rarity in nature but with unknown distributions of real occurrences. The ratio of singletons to doubletons within grid cells is higher in Africa and Oceania followed by South America and is quite low in North America and Eurasia.

From the rarity data among samples (occurrence-based rarity), we estimated that South America accounts for the highest total number of rare species (∼8,200) followed by Eurasia (∼6,100) and Africa (∼3,900). In Eurasia and North America, the percentage of species rare among grid cells was ∼43%, and it was <40% in the other continents, with the lowest value in South America (∼30%) (*SI Appendix*, Table S3). The ratio of singletons to doubletons among grid cells in North America (1.83) is the highest among continents; for all other continents, it is lower than 1.5. At a global scale, percentage abundance-based rarity is higher than occurrence-based rarity, while the ratio of singletons to doubletons shows the opposite trend. Since we were aware that the numbers (and proportions) of singletons/uniques and doubletons/duplicates (and their relative magnitudes) are very much a function not only of true rarity but also of sampling effort, in relation to true richness, we estimated all indices adjusting them for “true singletons/uniques” (*Materials and Methods*). However, our findings still confirm that most forests are likely to be dominated by just a few tree species (17) and include a long tail of rare species, which represents a consistent 30 to 40% of the overall tree richness in all continents. Although more species-rich regions (such as South America and Africa) have higher abundance-based rarity, North America and Eurasia (which contain more of a mix of biomes) showed higher occurrence-based rarity, and this finding could provide insights to better understand the biogeography of tree species on Earth.

Overall, almost a third of global tree richness on Earth is made up of rare species, which appear only once or twice in our samples. Thus, if the global forest system is dominated by a relatively modest number of abundant tree species, the global number of tree species strongly depends on those rarely detected (∼35%) (*SI Appendix*, Table S3) and undetected species (some large fraction of the ∼9,200 unobserved over the ∼73,300 estimated) (Table 1) (34). These results highlight the vulnerability of global forest biodiversity to anthropogenic changes, particularly land use and climate, because the survival of rare taxa is disproportionately threatened by these pressures (16–19). The higher threats for rare species are an important concern if we consider that their functions in ecosystems, the services they provide, and the ecoevolutionary patterns of these hyperrare tree species are still poorly known (16–20, 25).

### Comparisons across Continents.

**Fig. 4.**
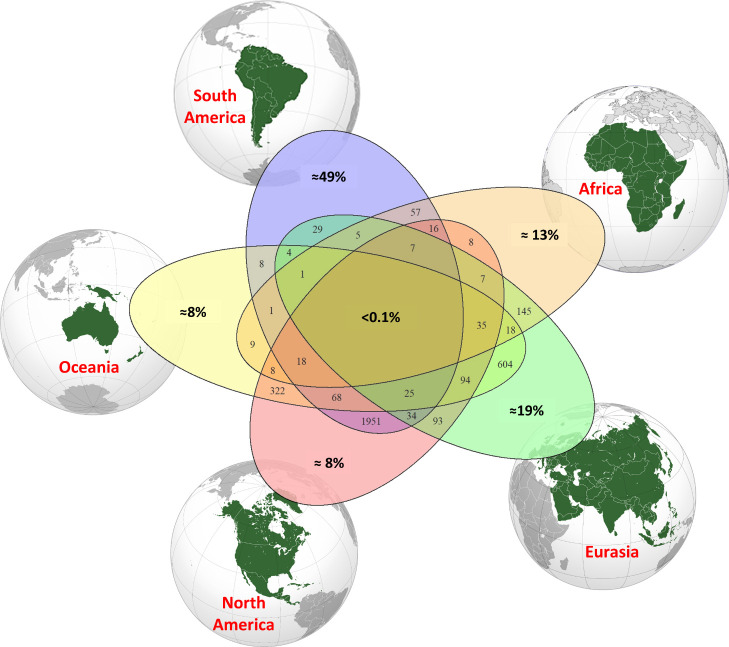
Species richness partitioning among continents. Estimates of the percentage of continental endemic (bold percentage values close to each continental map are based on the Chao2_adj_ estimator) (*Materials and Methods*) relative to the estimated richness per continent and shared species among continents (numbers in overlapping sets). In the center (bold percentage values at the intersection of all sets), the percentage of shared species among all five continents is shown.

To better understand the biogeography of richness patterns across land masses, we also estimated species turnover among continents (Fig. 4). Specifically, we combined the data of the five continents to obtain the values of estimated tree species richness in all 31 possible intersections (*Materials and Methods*). The two continents that share the highest estimated numbers of tree species are North and South America (Fig. 4), which is not surprising since these continents are interconnected by land (since about 3 Mya) in a region where nearby species-rich tropical forests occur on both continents. Consistent with this pattern, the second-highest number of shared species is between Eurasia and Oceania (Fig. 4), which had a geological continuity through the Southeast Asian archipelago that is another hotspot of tree diversity. Overall, other than the highest number of rare species, South America also shows the highest estimated percentage (49%) of continental endemic species (Fig. 4), while Eurasia and Africa account together for almost another 32% of unique tree species in the world. The percentage of shared species estimated among all five continents is lower than 0.1 (Fig. 4).

To summarize our main findings, we estimated that the absolute number of tree species on Earth is considerably higher than previously reported, with 14.3% more species than currently known to science (3). By establishing a quantitative benchmark, this information could contribute to tree and forest conservation efforts and the future discovery of new trees and associated species in certain parts of the world. For instance, considering that we estimated that about 31,100 tree species are expected in South America (Chao2_adj_ estimator) and those known to science are about 27,200 (Table 1), there might be about 3,900 tree species yet to be discovered in this continent, and most of them could be endemic (Fig. 4) and located in diversity hotspots of the Amazon basin and the Andes–Amazon interface. This makes forest conservation of paramount priority in South America, especially considering the current tropical forest crisis from anthropogenic impacts such as deforestation, fires, and climate change. Similar arguments can be made about the prioritization of conservation of tropical and subtropical forests on other continents given the considerable numbers of likely undiscovered species on each and their likely rarity. For example, there are likely high numbers of undiscovered species in Central America and in Southeast Asia.

This study accelerates our science by estimating global tree richness with a more extensive dataset and more advanced statistical methods than previous attempts. However, both the underlying data and Chao richness estimators and adjustments are imperfect. We recognize several methodological issues that might have potentially biased our estimates and/or contributed to uncertainty. The first involves the uneven and unrepresentative distribution of the sampling areas in the globe and within continents, which is an issue despite the high–sample coverage metrics that we used. The second involves the possibility that some species might have been misclassified due to misidentification, failure to update taxonomic name changes, and misspellings, which could reduce accuracy in estimates of species numbers (24, 33, 34). There is compelling evidence of errors in most biodiversity datasets due to the inclusion of false uniques (24). For example, if two botanists in different parts of the same forest region encounter the same species of rare and unfamiliar tree, they may identify it differently or use different synonyms to identify it, biasing the count of uniques and the estimators. Therefore, because of the likely discrepancy between the actual proportion of uniques in a sample and the observed unique count included in our datasets, we estimated the true number of unique species (28) and from this adjusted number, computed and focus on a more conservative estimate of global tree richness, which is ∼73,300 species (Chao2_adj_). There is also uncertainty about the accuracy of nonparametric estimators. Previous studies report that nonparametric estimators give lower values of tree species richness than parametric ones for the Amazon basin (34–36). However, our nonparametric estimate of tree species diversity in South American tropical forest biomes was higher than both parametric estimation and previous estimates in the Amazon (36). This might have resulted from previous studies being mainly based on Amazon lowlands, ignoring highlands. Thus, we examined sample completeness comparing continents but limiting their latitude to 23°N and S (tropical regions) (*SI Appendix*, Table S4). Results generally showed similar sample coverage at the grid-scale size used.

Future estimates of tree species richness in tropical, subtropical, and montane areas on all continents will be more accurate if an increased sample size is obtained (37), especially from areas poorly investigated. This begs the question on why South America alone could harbor >40% of all tree species. Compared with forest ecosystems on other continents, South America could have offered a larger continuous tropical forest area, a higher rate of speciation, a more robust mechanism of biodiversity maintenance, and reduced extinction rates [for instance, mild climates and the shortest period of human disturbance (39, 40)]. We also noticed that the SAC of South America continued to rise along the samples, whereas those of other continents start to level off, supporting the idea that undiscovered species numbers are likely high there, including in the Andean forests between 1,000- and 3,500-m altitude. A key challenge now is to install more plots in the Amazon-Andean transition zones, and to identify and monitor the trees within these plots.

Overall, our study points toward an estimated global tree richness (∼73,300) that is roughly 14% higher than numbers known today (3, 4), with many unknown species belonging to the tail of rare ones and often endemic to certain regions all across the globe. These results highlight the vulnerability of global tree species diversity to anthropogenic land use changes and to future climate (16–18). Losing regions of forest that contain these rare species will have direct and potentially long-lasting impacts on the global species diversity and their provisioning of ecosystem services (18–20). These results demonstrate both the lack of knowledge we still have about the tree species within our global forest systems and the value of approaches to help fill those gaps, which will be useful in providing fundamental insights about the diversity of life on our planet and its needed conservation.

## Materials and Methods

### Dataset and Sample Coverage.

We used the tree definition agreed on by IUCN's (International Union for Conservation of Nature) Global Tree Specialist Group (GTSG): “a woody plant with usually a single stem growing to a height of at least two meters, or if multi-stemmed, then at least one vertical stem five centimeters in diameter at breast height.” A tree inventory abundance dataset from 105,749 forest plots, ∼38 million stems of 28,192 species, distributed across all five continents was compiled from the GFBI (https://gfbinitiative.net/) database. For the Tonga and Niue data in the GFBI dataset, the original source was the New Zealand National Vegetation Survey Databank. For the estimation of the total number of tree species worldwide, we further compiled an independent occurrence dataset that we combined with the GFBI data. The occurrence-based dataset (hereafter, TREECHANGE) consists of taxonomy and location of >6 million tree individuals. Being a major data infrastructure itself, this dataset represents species occurrence information and encompasses a huge variety of data—from ground-sourced forest plot data (similar to the GFBI). Supported by a large body of collaborating institutions all over the world, this dataset features extensive global coverage and has been used across many large-scale studies (26). A limitation of the TREECHANGE dataset is that its underlying datasets do not have a coherent and consistent design and sampling scheme, but as described below, it complements the calculation of the estimated total number of tree species worldwide based on GFBI data. We extracted taxonomic data and associated geographic coordinates from five main data aggregators of species occurrences: the Global Biodiversity Information Facility [accessed through rgbif R package (41)], the public domain of the Botanical Information and Ecological Network v.3 [accessed through the BIEN R package (42)], the Latin American Seasonally Dry Tropical Forest Floristic Network [DRYFLOR (43)], the RAINBIO database (44), and the Atlas of Living Australia [ALA; accessed through the ALA4 R package (45)]. The species list was initially extracted from a world tree species checklist [GlobalTreeSearch (46)]. We checked for taxonomic correctness using the Taxonomic Name Resolution online tool (47), following a quality assessment and control of the data using the workflow outlined in ref. 26. This workflow minimized common errors associated with occurrence data (43). GlobalTreeSearch uses the tree definition agreed on by IUCN's GTSG above mentioned.

For abundance-based analyses, we used the GFBI tree species dataset (at its original plot size), whose samples cover a total area of more than 73,000 ha (*SI Appendix*, Table S1). Then, to perform occurrence-based estimations, we compiled a larger and more comprehensive global dataset with 100- × 100-km sampling units (∼1° grid cells) by combining the abundance-based data in the GFBI tree species dataset, which were converted in presence/absence occurrence data and pooled with the high-quality large occurrence-based TREECHANGE dataset. Globally, this yielded a dataset of 9,353 sampling units, with 696,063 occurrences. At the continental level, the combination of the two datasets to obtain a large occurrence-based dataset also yielded a number of sampling units somewhat comparable, in the sense of being a similar order of magnitude (Africa: 1,575; Eurasia: 2,896; North America: 2,418; South America: 1,461; Oceania: 1,003).

To ensure that our estimations of species richness were not biased by differences in sample coverage (e.g., an estimate of the total probability of occurrence of the species observed in the sample, taking into account species present but not detected) among continents, we estimated the inventory completeness (as defined by ref. 48) for the complete database and for each continent separately using the Chao–Shen sample coverage estimator (38, 48), which is a bias-reduced estimator of sample completeness:

$$C_{n}=1-\frac{f_{1}}{n}\left[\frac{\left(n-1\right)f_{1}}{\left(n-1\right)f_{1}+2f_{2}}\right],$$

where *f*_1_ and *f*_2_ are the numbers of singletons and doubletons (for abundance-based data) or the species occurred in only one (uniques) and in two (duplicates) 100- × 100-km (∼1°) samples (for occurrence-based data), respectively; *n* is the total number of individuals (for abundance-based data) or occurrences (for occurrence-based data) in the sample; and *C_n_* is the proportion of the total number of individuals (for abundance-based data) or occurrences (for occurrence-based data) in an assemblage (observed and not observed) that belong to the species represented in the sample (49, 50).

Because estimates of species richness can be strongly dependent on differences in inventory completeness, we checked whether sample coverage was similar in all five continents. Since all continents showed a similar proportion of sample coverage (all >94%), both from occurrence- (Table 1) and abundance-based data (*SI Appendix*, Table S1), we confirmed that our global estimate—based on global sample coverage of 96.44% (occurrence data) and 99.97% (abundance data)—was not disproportionately influenced by any specific continent. However, the slightly lower occurrence-based sample coverage of South America and Eurasia, with 95 and 94.26%, respectively, and the clustered distribution of some plots could explain the nonsaturating trend of their accumulation curves compared with the other continents (Fig. 2*B*). We also note that sample completeness at finer scales would be lower in all continents.

We selected the continental scale for our estimates, together with the common study frames of biomes (51), because nonparametric species richness estimators perform better when samples are collected in a continuous incremental area without relevant landmass separation such as oceans (31–33).

For instance, working at a global biome-level only would ensure that the current climatic conditions are similar, but this approach to estimate species richness, taken alone, would reduce the information implied in the estimates because they would be affected by several factors. 1) Within each across-ocean biome, there are still important ecological and evolutionary differences that would affect the estimates at the global biome level [in fact, conventional levels of ecological hierarchical organization are not scale dependent (52), whereas species richness estimates are]. 2) With nonparametric estimates based on SACs, it is better to ensure a continuity of sampled areas (e.g., continuous terrestrial lands) (53, 54). 3) The ecological conditions that have shaped the evolutionary patterns (phylogeny and diversity) of tree species on Earth were much different when continents were conglomerate in Pangea (55) and then slowly shifted away (i.e., during this long geological time, biomes were much different to current ones) (56–58).

Therefore, other than estimating global tree species richness at a global biome level (Fig. 3 and *SI Appendix*, Table S2), we analyzed continental richness to also account for evolutionary changes in response to the biome main variables (latitude, climate, solar radiation, etc.), which shaped current tree diversity. Adding the figures at a continental (and a continental biome) level, we ensured that our estimates are based on the 135-My biogeographical and temporal continuity of the five main vegetated landmasses, which is an implied assumption of the estimators. This approach also allows a better discussion of the results for species turnover among continents (Fig. 4), which might be a result of their connections in Laurasia and Gondwana and the following continental drift.

### Species Richness Estimators.

We initially computed a parametric estimate of species richness on the abundance-based data for 28,192 species from the GFBI dataset (*SI Appendix*, Table S1). In particular, we considered the Fisher’s *α* for abundance data (calculated from http://groundvegetationdb-web.com/ground_veg/home/diversity_index).

We found that the abundance-based Fisher’s *α* underestimated the absolute species richness because our global (*SI Appendix*, Table S1) Fisher estimate was close but lower than the observed number of species in our occurrence-based dataset (64,100 from GFBI + TREECHANGE). Because this parametric estimator assumes a log-series distribution of abundances (59), we performed a goodness-of-fit test and evaluated it with a Kolmogorov–Smirnov test of whether our global and abundance data fit a log-series distribution. Since all datasets (global: *D* = 0.1, *P* = 1; Africa: *D* = 0.1, *P* = 1; Eurasia: *D* = 0.5, *P* = 0.17; North America: *D* = 0.2, *P* = 0.99; Oceania: *D* = 0.2, *P* = 0.99; South America: *D* = 0.2, *P* = 0.99) follow a log-series distribution, we calculated the *α*-values. At a global level, we obtained a Fisher’s *α*-value of 3,040 (*SI Appendix*, Table S1).

We used this value and the most recent estimates on the global number of trees by Crowther et al. (60) to estimate the global number of species from Fisher’s classical equation (61):

$$S=\alpha \text{ln}\;\left(1+\frac{N}{\alpha }\right),$$

where *N* is the total number of trees and *α* is the Fisher’s *α*-parameter. This yielded an estimate of 62,624 to 62,915 species (lower–upper bootstrap 95% CI) from the 3.04 ± 0.19 × 10^12^ (±95% CI) global tree stems calculated by Crowther et al. (60). Although Fisher’s parametric approach stands on the very strict assumption of infinite log-series species abundance distributions, giving rise to overestimation of hyperrarity (62, 63), it estimated slightly less than the observed number of species in our occurrence-based dataset (using the *α*-value derived from our abundance-based dataset). We thus did not further employ this estimate. Instead, with the larger occurrence dataset composed of GFBI (converted to presence/absence) and TREECHANGE data, we then calculated the Chao2 index, which is a lower-bound estimator and considered one of the most reliable and less affected by bias among all nonparametric indices (27, 64–66). The values of the estimators from the samples to plot the curves shown in Fig. 2 were randomized, interpolated, and extrapolated with the package iNext in R (67).

The Chao2 estimator (bias corrected) is calculated by the following formula:

$$\mathrm{Chao}2=S_{\mathrm{obs}}+\left(\frac{m-1}{m}\right)\left(\frac{Q_{1}(Q_{1}-1)}{2(Q_{2}+1)}\right),$$

where *S_obs_* are the actual numbers of species observed in the samples (*m*) and *Q*_1_ and *Q*_2_ are the species that appear in only one (unique) and two (duplicate) sampling units, respectively (27, 29). The 95% CI (CI bias corrected) of this index can be calculated by the formula

$$\text{Lower}\;95\%\;\text{Bound}=S_{\mathrm{obs}}+\;T\text{/}K;\;\text{Upper}\;95\%\;\text{Bound}\;=S_{\mathrm{obs}}+\;TK,$$

$$\text{where}\;T=\mathrm{Chao}2-S_{\mathrm{obs}}\text{and}\;K=\text{exp}\left\{1.96{\left[\text{log}\left(1+\frac{\hat{\mathrm{var}}({\hat{S}}_{\mathrm{Chao}2})}{T^{2}}\right)\right]}^{1/2}\right\}.$$

This estimation yielded a global value of 89,147 ± 1,101.5 species (Chao2 ± 95% CI) (Table 1). We are well aware that some studies provide different preferred estimators (68–70). However, many analyses, including simulation-based experiments, encourage the use of Chao2 to minimize bias (a summary is in ref. 71). This is the reason we considered the Chao2 index (based on occurrence data) our more useful estimator. Nonetheless, this estimator is sensitive to accurate quantification of the numbers of uniques and duplicates, and it is known that there are problems with false uniques in forest species richness datasets (24). Our Chao2 values may, thus, represent an overestimate to the degree that tree species recorded in only one sampling unit have been mistakenly identified as unique. Therefore, to check the reliability of our nonparametric estimates, we calculated the true number of uniques $\hat{(Q_{1}})$ (28) in each continent and at a global scale to understand whether our values were influenced by the number of “falsely unique species.” This estimation of the true number of uniques is calculated with the formula adapted from ref. 28 for incidence-based data:

$$\hat{Q_{1}}=\left(\frac{T-1}{T}\right)\frac{2Q_{2}^{2}}{3Q_{3}}+\left(\frac{T-1}{T}\right)2Q_{2}\left(\frac{Q_{2}}{2Q_{3}}-\frac{Q_{3}}{4Q_{4}}\right),$$

where $\hat{Q_{1}}$ is the estimated true number of uniques; *T* is the number of sampling units (map cells); and *Q*_2_, *Q*_3_, and *Q*_4_ are observed duplicates, triplicates, and quadruplicates.

At the global level, the estimate of the true number of uniques is 13,162 compared with the observed 24,768. At the continental level, the number of estimated uniques was much lower than the observed one in South America (4,888 vs. 13,110) and somewhat lower in Eurasia (3,424 vs. 5,806), Africa (2,192 vs. 3,466), and Oceania (1,444 vs. 2,208), but it was slightly higher in North America (2,460 vs. 2,360). We then used the adjusted number of uniques in the Chao2 equation (see above) to calculate the Chao2_adj_ estimates, ${\hat{S}}_{\mathrm{adjChao}2}$ (27–29).

We also calculated tree species rarity at continental and global scales for abundance (abundance-based rarity; i.e., based on the number of adjusted singletons [*S1*] and doubletons [*S2*]) and occurrence (occurrence-based rarity; i.e., based on the adjusted number of unique species and the number of duplicate ones). We defined the number of rare species as the sum of adjusted singletons and doubletons. We also computed an index of rarity importance using our occurrence-based dataset as the proportion of rare species over total richness and an *S1_adjusted_/S2* ratio, which is the proportion of singletons over doubletons.

### Continental Biodiversity Partitioning.

We estimated the number of species shared among continents and unique to each continent using the Chao2 estimator (Fig. 4) from the occurrence-based data, and we represented them in a Venn diagram. We combined the observations of species richness for the five continents (*n* = 5) in all possible 2^5^ − 1 = 31 combinations.

First, we calculated asymptotic species richness (Chao2) from occurrences observed in each continent; then, we intersected (creating a unique presence/absence binary entry for each species) the observed occurrences per each pair, triplet, quadruplet, and all five of continents (obtaining the occurrences of all the observed species in each combination of continents) and calculated the asymptotic species richness (Chao2) per each pair, triplet, quadruplet, and quintuplet continents. Therefore, a total of 31 estimates were obtained by the Chao2 index and plotted in a Venn diagram with the R package VennDiagram (72).

Additional cross-checks of the data pooling approach and *q1/q2* relationship are in *SI Appendix*, *SI Methods*.

## Supplementary Material

Supplementary File

## Data Availability

Anonymized numeric data have been deposited in GFBI, https://gfbinitiative.net/data/.
