# Supplementary material for: The number of tree species on Earth
Source: Proc Natl Acad Sci U S A. 2022 Jan 31;119(6):e2115329119. doi: 10.1073/pnas.2115329119 (PMC8833151; doi:10.1073/pnas.2115329119)
Supplement: Supplementary File [file pnas.2115329119.sapp.pdf]

Supporting Information (SI)

<u>Supplementary Methods</u>

<u>Additional cross-checks of the data pooling approach and *q1/q2* relationship (see SI Appendix)</u>

We conducted additional cross-checks to evaluate if the sample size, homogeneity and samples distribution may have affected our estimates. One of the main reasons for doing this is that Chao's estimators ($S_{obs}+S_{exp}$) depend on f_1 (singletons in abundance-based data) or q_1 (uniques in occurrence-based data) and f_2 (doubletons) or q_2 (duplicates) to derive the expected number of species (S_{exp}, which are added to the *S* observed ones). In our abundance-based (GFBI) dataset, we had (for instance, in South America) f_1=5,739 and f_2=2,320 for abundance-based data (i.e. f_1/f_2=2.5 and S_{exp}=7,098) but q_1=12,349 and q_2=1,594 for occurrence-based data (i.e. q_1/q_2=7,7 and S_{exp}=47,834). To account for this increased proportion from f_1/f_2 to q_1/q_2 (i.e. from 2.5 to 7.7) which significantly increases the estimates of ≈40k additional species, we proceeded in a 3-step cross-check of the correctness and potential bias of our data pooling approach and q_1/q_2 relationship:

1) Checking the relationship between grid size and unique (q_1) and duplicate (q_2) species

In this analysis, we used the data from the GFBI abundance database (transformed to occurrence data) and the occurrence data from the TREECHANGE database (occurrence based).
We studied the behavior of q_1 and q_2 reducing the grid cell size from 1° to 0.01° in the most diverse continents, South America, Africa and Oceania (Table S5).
We found a slight decrease of q_1 and q_2 in all of the 3 continents but the relationship between q_1 and q_2 (thus the *S* expected from Chao's eq.) remains approximately constant. This shows that the variation in grid cell size does not affect the estimation of *S* expected.
As an additional check, to account for "false unique species" due to cells with a low number of species detected, from the same analysis we removed all grid cells with less than 4 observed species (Table S6).
Also in this case, we found a slight decrease of q_1 and q_2 in all of the 3 continents but the relationship between q_1 and q_2 (thus the *S* expected from Chao's eq.) remains approximately constant even after removing the cells with less than 4 species. Some small differences appear in South America between the two approaches (Figure S1) but they are limited to less than 1000 species at the lowest grid cell size (<0.02°). This confirms, as shown in the previous check, that the grid size does not affect the estimation of *S* expected.

2) Checking the Comparison between unique (q_1) and duplicate (q_2) species and the data

We then compared q_1 and q_2 in South America (the most species-rich continent) by modifying the datasets to understand if either one of the two datasets or their combination may have influenced the estimates. Since, from the previous step, we found minimal influence of the grid cell size on the estimates, we used a standard 1° grid cell resolution for this analysis.
We first determined in which cells there are GFBI and TREECHANGE data (Figure S2). Then we extracted q_1 and q_2 from the single and combined datasets in those cells that both contained GFBI and TREECHANGE data (Table S7). This analysis allowed us to find that the big difference between q_1 and q_2 is in the GFBI dataset (once it is converted to occurrence-data). One reason of this can be found in the unequal distribution (clustered) of GFBI plots in specific areas of South America although this does not completely explain why there are ≈12k species that are found only in one plot (uniques) of the whole dataset of this continent.

Therefore, to disentangle the reasons why we have a high proportion of q_1/q_2 in the GFBI (occurrence) data, we proceed with the step 3.

3) Checking q_1 and q_2 species in South America at a GFBI plot resolution

We calculated q_1 and q_2 at GFBI plots resolution (and no longer at 1° grid cell resolution). However, to avoid losing too many species from the data, we added the nearest TREECHANGE occurrences into the GFBI plots (i.e. we added the TREECHANGE occurrences that were within the 1°cell to each GFBI plot in which the GFBI plot was located; see Figure 2). This was done at the continent level for South America and at the biome level in South America.
At continental level (South America) we found: unique species (q_1) = 10,316 and duplicated species (q_2) = 2,027. Therefore, even at the GFBI plot scale the proportion of q_1/q_2 is similar to that at 1° degree cell. This shows that our estimates are not influenced by the change of sample size from GFBI plot scale (smaller plots) to 1° degree cell (bigger cells).
As a final cross-check, we tried to understand if the high value of q1 (species found in only one GFBI plot) was due to the uneven distribution of plots in different biomes (assuming that the plots in the same biome should share more species). We, therefore, calculated q_1 and q_2 at the biome level in South America (Table S8).
We found that, even within each biome (particularly in Biome 1 – tropical forests - which account for the highest richness of South America) the proportion q_1/q_2 remains high and does not depend on the uneven distribution of plots in different biomes.
After these additional cross-checks, we concluded that our estimates are not biased by artifacts due to resampling, data distribution or lack of homogeneity among samples but depend on the pure nature of the available GFBI data. Thus, the only possibility to account for the "false" uniques in our dataset was to estimate (at both the continental and global level) the true number of uniques.

Supplementary tables and figures

Table S1. Fisher's alpha estimated at continental and global scales from abundance-based (GFBI) data only

	Africa	Eurasia	North America	Oceania	South America	*Global*
N. samples	1,374	64,086	30,914	4,133	5,242	105,749
Area (sampled; ha)	435.6	14,757.2	54,918.0	688.1	2,905.9	73,704.8
Sample coverage (%)	99.43	99.98	99.99	99.81	99.68	99.97
Fisher's alpha (α-value)	640	484	157	356	2,579	3,040

Table S2. Observed (Obs. S) and estimated (Chao2 adjusted) tree species richness, and sample size (numbers of grid cells, *n*) at continental biome-level, for biomes with at least 500 grid cells globally. q1 is the number of unique species, q2 is the number of duplicate species, qi adj is the adjusted number of uniques, 95% CI adj are the lower and upper bounds of the confidence interval for the Chao2 adjusted value

	**Africa**								**Eurasia**								**North America**								**South America**								**Oceania**							
**Forest Biome**	Obs. S	n	q1	q2	q1 adj	Chao2 adj	lower 95% C.I. adj	upper 95% C.I. adj	Obs. S	n	q1	q2	q1 adj	Chao2 adj	lower 95% C.I. adj	upper 95% C.I. adj	Obs. S	n	q1	q2	q1 adj	Chao2 adj	lower 95% C.I. adj	upper 95% C.I. adj	Obs. S	n	q1	q2	q1 adj	Chao2 adj	lower 95% C.I. adj	upper 95% C.I. adj	Obs. S	n	q1	q2	q1 adj	Chao2 adj	lower 95% C.I. adj	upper 95% C.I. adj
**Tropical and subtropical moist broadleaf forests**	6,865	515	1990	1225	2314	9,044	8,870	9,215	10,043	709	4,157	2,093	4,370	14,595	14,349	14,838	5,941	175	1,825	973	2,601	9,394	9,283	9,455	16,677	922	4,868	2,850	6,101	23,197	22,913	23,282	3,911	125	1,827	794	1,785	5,898	5,766	6,065
**Tropical and subtropical dry broadleaf forests**	1,126	32	472	297	913	2,479	2,328	2,623	784	103	608	126	783	3,173	2,950	3,389	3,147	177	1,210	368	586	3,608	3,447	3,721	3,707	241	1,513	547	1,328	5,309	4,957	5,426	629	19	482	87	238	934	934	928
**Temperate broadleaf and mixed forests**	29	3	22	7	52	140	129	157	1,428	915	479	207	405	1,820	1,725	1,932	543	400	60	45	93	636	592	683	95	57	23	8	0	95	85	128	1,408	161	191	145	327	1,770	1,719	1,806
**Temperate conifer forests**	56	9	28	7	0	56	34	74	1,363	255	478	206	314	1,599	1,502	1,688	709	461	109	46	108	832	800	869	0	0	0	0	0	0	0	0	0	0	0	0	0	0	0	0
**Boreal forests/taiga**	0	0	0	0	0	0	0	0	108	335	26	8	18	126	101	154	128	742	19	9	17	142	118	159	0	0	0	0	0	0	0	0	0	0	0	0	0	0	0	0
**Tropical and subtropical grasslands, savannas and shrublands**	3,257	954	1075	528	972	4,147	4,011	4,259	189	15	129	32	57	235	193	272	206	24	41	40	65	255	233	287	4,976	433	2,079	634	1,486	6,709	6,593	6,112	1,764	284	373	190	291	1,984	1,943	2,036
**Temperate grasslands, savannas and shrublands**	0	0	0	0	0	0	0	0	107	102	56	24	55	167	163	182	401	323	58	28	67	476	451	525	157	78	61	28	38	181	174	185	410	102	83	61	110	506	486	533
**Tundra**	0	0	0	0	0	0	0	0	65	109	15	3	0	65	59	76	22	178	6	1	0	22	8	39	0	0	0	0	0	0	0	0	5	3	3	2	4	6	6	6
**Mediterranean forests, woodlands and scrub**	418	86	83	78	177	613	587	639	273	239	40	27	66	348	329	373	285	44	47	29	40	310	290	337	52	28	30	11	61	198	191	211	784	146	92	78	133	895	876	903
**Deserts and Xeric shrublands**	1,026	242	355	181	333	1,328	1,201	1,455	328	167	174	76	189	558	474	638	1,425	369	471	193	445	1,932	1,878	1,983	2,120	160	730	317	548	2,588	2,486	2,697	660	375	121	78	161	823	789	851

Table S3. Tree species rarity at continental and global scales for abundance-based ("abundance-based rarity") and occurrence-based ("occurrence-based rarity") data. The adjusted number of singletons (or uniques) is calculated from the true number of singletons/uniques (see Methods). Rare species is calculated as the sum of singletons (adjusted) and doubletons; % rarity is the proportion of rare species over total richness; $S1_{adjusted}/S2$ is the proportion of singletons/uniques adjusted over doubletons/duplicates.

	Continent	Total richness observed	Total individuals (or occurrences) observed	Singletons (or unique) observed	Singletons (or unique) adjusted	Doubletons (or duplicate) observed	**Rare species adjusted**	**% rarity (rare adj./total)**	$S1_{adj}/S2$ **ratio**
**Abundance-based rarity**	Africa	3,528	157,887	901	880	468	**1,348**	**38.21**	**1.88**
	Oceania	2,407	306,782	581	529	287	**914**	**33.90**	**1.84**
	Eurasia	4,564	6,028,218	1,041	627	468	**997**	**23.99**	**1.34**
	North America	1,875	24,058,117	248	171	156	**327**	**17.44**	**1.10**
	South America	16,877	2,905.938	5,739	3,941	2,320	**6,261**	**37.10**	**1.70**
	Global	28,188	32,341,275	8,179	5,840	3,529	**9,369**	**33.24**	**1.65**
**Occurrence-based rarity**	Africa	10,441	86,708	3,466	2,192	1,672	**3,864**	**37.01**	**1.31**
	Oceania	7,175	83,694	2,208	1,444	981	**2,425**	**33.80**	**1.47**
	Eurasia	14,073	101,142	5,806	3,424	2,673	**6,097**	**43.32**	**1.28**
	North America	8,888	162,380	2,360	2,460	1,347	**3,807**	**42.83**	**1.83**
	South America	27,574	262,139	13,110	4,888	3,372	**8,260**	**29.96**	**1.45**
	Global	64,088	696,063	24,768	1,3162	9,426	**22,588**	**35.25**	**1.40**

Table S4. Occurrence data in each continent selected between latitude 23°N and 23°S to evaluate the differences in the sample coverage only for plots in the tropical strip of each landmass

Sample Coverage - Tropical Areas (23°N-23°S)	
Continent	%SC
Africa	95.16%
Eurasia	82.47%
Oceania	94.46%
North America	91.62%
South America	94.60%

Table S5. Relationship between grid size (in degrees. 1º ~ 111 km) and unique (q_1) and duplicates (q_2) species. OC: Oceania; AF: Africa; SA: South America.

Grid size	*OC (q_1)*	*OC (q_2)*	*AF (q_1)*	*AF (q_2)*	*SA (q_1)*	*SA (q_2)*
1	2417	1080	3469	1679	13403	3445
0.9	2300	1054	3442	1602	13217	3466
0.8	2245	964	3328	1593	13213	3329
0.7	2169	1007	3264	1546	13112	3315
0.6	2138	926	3196	1553	12952	3282
0.5	2076	937	3066	1515	12825	3203
0.4	2019	874	2992	1465	12690	3158
0.3	1899	865	2838	1429	12578	2991
0.2	1827	774	2774	1312	12329	2958
0.1	1696	699	2599	1246	12072	2750
0.01	1494	580	2352	1046	11579	2479

Table S6. Relationship between grid size (in degrees. 1º ~ 111 km) and unique (q_1) and duplicate (q_2) species. OC: Oceania; AF: Africa; SA: South America. In this table we removed grid cells with less than 4 observed species.

Grid size	*OC (q_1)*	*OC (q_2)*	*AF (q_1)*	*AF (q_2)*	*SA (q_1)*	*SA (q_2)*
1	2411	1082	3480	1672	13411	3437
0.9	2290	1053	3445	1604	13221	3469
0.8	2243	961	3338	1590	13347	3302
0.7	2171	999	3281	1536	13256	3301
0.6	2130	926	3206	1550	13139	3275
0.5	2066	938	3083	1508	12843	3201
0.4	2010	875	3010	1470	12977	3159
0.3	1884	865	2864	1435	12873	3021
0.2	1820	782	2811	1311	12683	2985
0.1	1691	684	2679	1250	12678	2888
0.01	1631	605	2896	1075	12783	2759

Table S7. q_1 and q_2 according from the dark and light red cells (see Figure S4).

Dataset	*q1*	*q2*	*Total n° of species*
GFBI	12349	1594	16880
TREECHANGE	4383	2496	13263
GFBI ∪ TREECHANGE	14205	3259	25387
GFBI ∩ TREECHANGE	234	546	4756
GFBI - TREECHANGE	10389	835	12124
TREECHANGE - GFBI	3562	1878	8507

Table S8. Calculation of q_1 and q_2 in all biomes of South America

South America biomes	*q1*	*q2*
Biome 14	148	8
Biome 13	209	159
Biome 12	0	1
Biome 10	0	0
Biome 8	0	0
Biome 7	280	696
Biome 4	20	0
Biome 3	353	22
Biome 2	2063	408
Biome 1	9461	1968

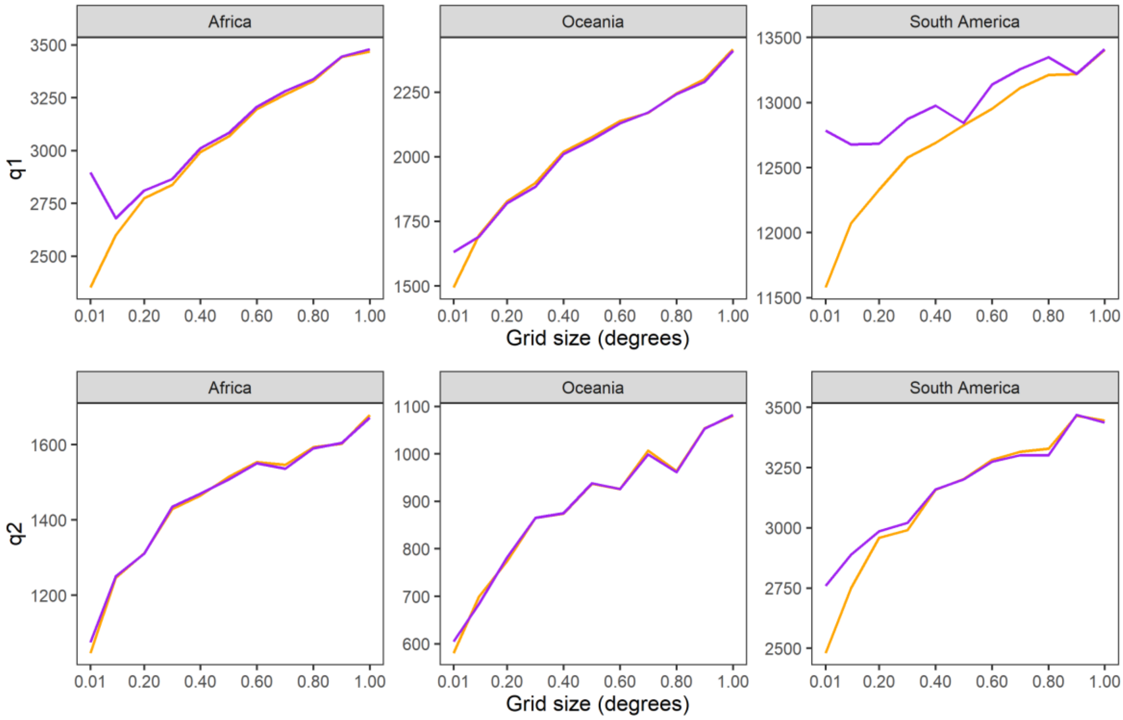

Figure S1. Graphical representation of Tables S5 and S6. The orange line corresponds to the Table S5 (without removal of the grid cells with low number of species) and purple line corresponds to the Table S6 (removing grid cells with less than 4 observed species).

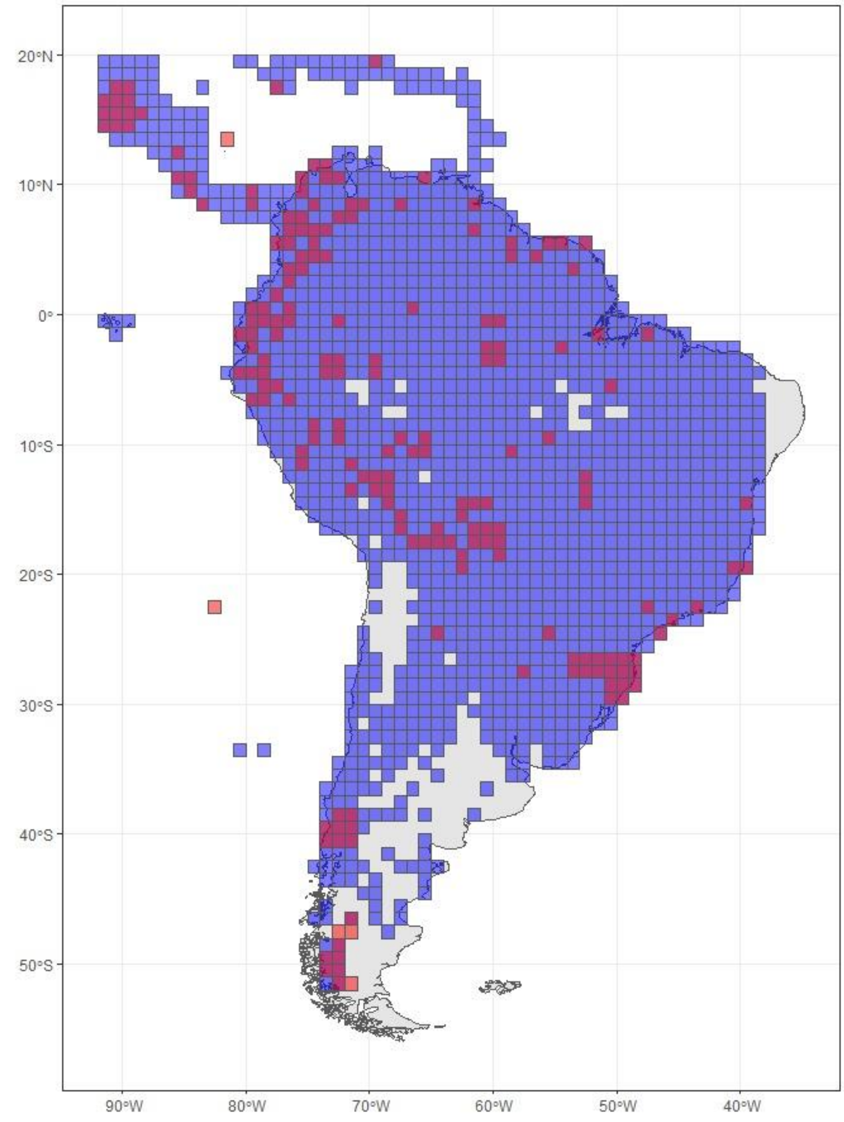

Figure S2. Location of GFBI and TREECHANGE data in a 1º resolution grid. Blue cells have only TREECHANGE data; dark red cells have both GFBI and TREECHANGE data; light red cells have only GFBI data.
